# Drug resistance in cancer: molecular evolution and compensatory proliferation

**DOI:** 10.18632/oncotarget.7459

**Published:** 2016-02-17

**Authors:** Ran Friedman

**Affiliations:** ^1^ Department of Chemistry and Biomedical Sciences, Linnæus University, Kalmar, Sweden

**Keywords:** nearly neutral theory, neoplasm, leukemia, bladder cancer, somatic mutations

## Abstract

Targeted therapies have revolutionized cancer treatment. Unfortunately, their success is limited due to the development of drug resistance within the tumor, which is an evolutionary process. Understanding how drug resistance evolves is a prerequisite to a better success of targeted therapies. Resistance is usually explained as a response to evolutionary pressure imposed by treatment. Thus, evolutionary understanding can and should be used in the design and treatment of cancer. In this article, drug-resistance to targeted therapies is reviewed from an evolutionary standpoint. The concept of apoptosis-induced compensatory proliferation (AICP) is developed. It is shown that AICP helps to explain some of the phenomena that are observed experimentally in cancers. Finally, potential drug targets are suggested in light of AICP.

## TARGETED TREATMENT

One of the most important breakthroughs in cancer treatment was the development of a chemical compound called imatinib mesylate, which is used in cancer therapy since 2001 [1]. Imatinib is an inhibitor of the Abl1 kinase. Glivec^®^, the drug which contains imatinib as the active compound, has been described as a ‘magic bullet’ [2]: a treatment that works directly against a given target without causing harm elsewhere. Owing to the vast success of imatinib, several other kinase inhibitors have since been developed and applied in the clinic. Overall, at the time of writing, 30 kinase inhibitors have been approved by the FDA to treat 15 different diseases.

Proteins other than kinases are also targeted by non-cytotoxic oncological drugs. Examples include the PSMB5 protein, which is part of the proteasome; and sex hormone receptors that are important drug targets in breast and prostate cancers. Drug targets of targeted therapies are proteins or pathways that acquire abnormal pathogenic properties [3]. Immunotherapy, which works by activating the body's own immune system against cancer, is another form of targeted therapy. Immunotherapy drugs are antibodies that work by inactivating proteins that down-regulate cytotoxic responses against cancer cells [4]. Immunological drugs appear to be very effective against some types of cancer including among others chronic lymphocytic leukemia, melanoma and non-small cell lung cancer.

## RESISTANCE TO TARGETED TREATMENT

Unfortunately, the promise of targeted treatment is quite often offset by the development of drug resistance [5, 6]. Resistance develops gradually within the population of tumor cells. When resistant cells dominate the population of the tumor, the disease ceases to react to medications that were once very potent. Drug resistance can have many forms. These include mutations in the drug target that prevent the binding of the drug [7], over-expression of proteins that compensate for the loss of the drug target [8], and activation of redundant biological feedback mechanisms [9, 10]. Once resistance emerges, the cancer is much more difficult to treat. In some cases, it is possible to switch to an inhibitor with a better resistance profile [11]. However resistance may also develop against the novel therapy. Immunotherapy, which is often portrayed in the popular media as the most promising strategy in contemporary drug design against cancer, (e.g., [12, 13]), is also susceptible to resistance [14]. Antiangiogenic therapy is also subject to drug resistance, through multiple mechanisms [15]. In fact, it may even select for metastatic clones to avoid hypoxia, thus making the cancer more violent [16].

**Figure 1:**
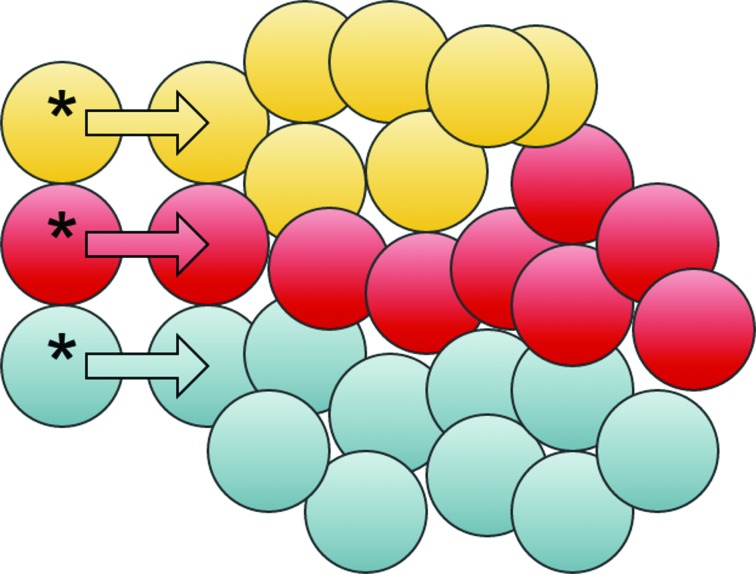
Clonal evolution Cancer stem cells gradually acquire mutations and become distinct. Each distinct cancer stem cell (cells marked with an asterix) replicates and generate cells that differentiate, leading to the formation of clones, illustrated here by different colors. The clones may have different properties with respect to their ability to survive under different sorts of pressure: treatment, hypoxia, nutrient shortage etc.

A key element in the understanding of resistance mechanisms is that of clonal evolution [17, 18] within the concept of population genetics [19, 20]. Cancer cells are genetically unstable. They acquire additional mutations and karyotypic modifications, that are inherited to daughter cells. Clonal evolution gradually leads to aggressiveness of the cancer and resistance to treatment. Various tumor clones have different capabilities to proliferate in the absence or presence of drugs (Figure 1), making the genetic landscape of the tumor clones highly dynamic [21]. Cancer has been described as a ‘moving target’ as the population of cells constantly shifts during therapy [22]. A better understanding of the evolutionary process that underlies drug resistance is needed for avoiding or postponing the emergence of resistance. This can be done e.g., *via* planning dosing schedules [23], using a combination of drugs [24] or through the design of medicines that would be more resilient to drug resistance [25].

## CANCER DRUG RESISTANCE AND POPULATION GENETICS

### Resistance mutations

The evolutionary dynamics of drug resistance can be reasoned by applying population genetics models. The drug target is often a protein or an enzyme that have been evolved to carry out its biological function(s). A kinase, for example, binds and phosphorylates specific proteins. There is often an intricate control, i.e., the drug target is activated by certain interactions, whereas it activates others. Driver mutations can make a protein constitutively active, by relieving it from the ability to be controlled. Once targeted by a drug though, the protein is incapable to carry out a function that is essential for the tumor. A resistance mutation recovers the function of the drug target even in the presence of the drug.

It is possible to divide resistance mutations into two groups. The first are mutations that interfere with the binding of the drug. The second are mutations that make the drug target perform their biological function more efficiently, so that those proteins that escape the drug make the tumor resistant. Given that the drug target has been evolved for so long to carry out its function in a way that is most beneficial for the organism, mutations of the first type are likely to be more common.

**Figure 2:**
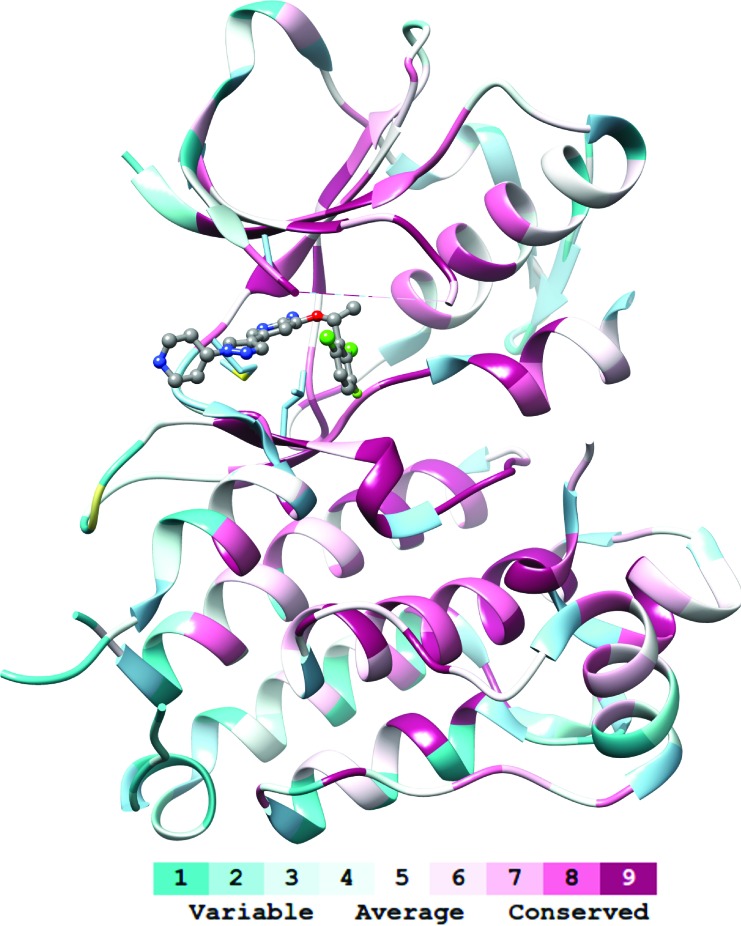
Conservation at the drug targets The drug targets (here: the kinase domain of anaplastic lymphoma kinase, ALK) are usually proteins whose function is crucial to the tumor cells. Such proteins include many residues where modifications are limited or unlikely to be tolerated (conserved residues, see the color code). Even at less conserved residues, random mutations can lead to destabilization of the structure or interfere with the biological activity. Under strong selective pressure on the tumor due to drugs (here: crizotinib, represented with balls-and-sticks), some mutations can become beneficial and lead to improved fitness. Evolutionary conservation was calculated with Consurf [83]. Molecular graphics and analyses were performed with the UCSF Chimera package [84]. UCSF Chimera is developed by the Resource for Biocomputing, Visualization, and Informatics at the University of California, San Francisco (supported by NIGMS P41-GM103311).

Mutations that interfere with drug binding do so by altering the active domain of the protein (e.g., the catalytic domain of an enzyme, Figure 2) directly or indirectly. Direct interference happens by altering residues at the binding site. Indirect interactions modify the dynamics of the protein, e.g., by destabilizing an inactive state that binds to the drug. In both cases, the effects of the mutations are not local to the drug, but can also alter the dynamics and hence efficiency of the whole protein. For this reason, only a relatively small subset of residues can in fact be mutated [25], even when dozens of resistance mutations can exist for the same protein [11].

### Definition of the fitness of tumors

The evolutionary concept of fitness refers to the ability of an organism to survive and reproduce (i.e., contribute genes to the next generation) [26]. This definition should be adjusted to be suitable to discuss tumors. Therefore, instead of discussing fitness over generations (Darwinian fitness), we refer to the Malthusian fitness, which is a measure of fitness over a small period of time [27]:

$$m=\frac{\text{ln}\left(N_{t}/N_{0}\right)}{t}$$

Here, *N_t_* and *N_0_* represent the number of individuals (cells) in the population initially and at time *t*. We can thus estimate the difference in the fitness of two populations (here: tumor clones) by their growth at a particular time frame. Tumor clones with large positive *m* will grow faster than others, whereas tumor clones with negative Malthusian fitness will shrink. Note that *m* is not a static constant. Rather, the Malthusian fitness, and the rate of tumor proliferation, depend on the genetic population and on the type of treatment (if any). Targeted therapy initially results in negative values of *m*, which makes the tumor shrink (at least somewhat). Drug resistance will set the value of the Malthusian fitness back to positive.

### Pre-existing or acquired resistance

Due to many different factors, the mutation rate in cancer cells is higher than in normal (non-tumor) cells. Furthermore, it can vary by as much as three orders of magnitudes between cancers and even patients [28]. In chronic cancers, the tumor may grow for years before it is treated, giving ample time for resistance mutations to emerge and get fixed within the population of tumor cells prior to the beginning of treatment. If the resistance mutation is neutral in all other aspects, i.e., it does not affect the fitness of the tumor prior to treatment, its probability of fixation is proportional to its proportion in the population, i.e., the earlier it emerges within the tumor the higher the chance that it would get fixed. In general, the probability of fixation of a mutant depends on the product *Ns* where *N* is the effective population size (number of cells capable of reproduction) and *s* is the selection coefficient, which is zero for neutral mutations, negative for mutations that lower the fitness and positive for those that increase the fitness. Quite often, slightly deleterious mutations (*Ns* close to zero but negative) get fixed in the population. The smaller is the effective population size, the more likely it is for deleterious mutations to get fixed. Thus, mutations that lead to resistance can be present in the tumor prior to treatment, even if they reduce the fitness of the tumor, as long as they emerge early enough. Once the treatment begins, the selection coefficient of the mutants increase at once because the cells that carry the mutation are drug resistant, whereas other cells are not.

In contrast to pre-existing resistance, acquired resistance means that, upon treatment, a mutation emerges that carries a selection benefit (positive selection coefficient) to the treated population of tumor cells. Such mutations are likely to be fixed in the population of the tumor cells. The longer the treatment, the higher the chance that a resistance mutation will emerge in this way. A more aggressive treatment (e.g., a higher dose of the drug) has two contradicting effects on the development of acquired resistance. On the one hand, it reduces the Malthusian fitness of sensitive clones and thus selects for resistance clones. On the other hand, it reduces the overall size of the tumor and thereby presumably also the effective population size, thus leading to a smaller population of cells that can mutate and acquire *de novo* resistance.

The discussion about pre-existing *versus* acquired resistance is not only of a theoretical nature. If a resistance mutation is pre-existing, tumor clones that carry this mutation will quickly be selected. The more aggressive the treatment, the more likely it is that such mutations will dominate the tumor cell population because their selection coefficient is higher. If, on the other hand, mutations are acquired upon treatment, the more aggressive the treatment the faster the tumor recedes and the less likely it is for such mutations to emerge. Note, however, that if a resistance mutation does emerge in a smaller population its probability to be fixed is higher.

Experimental evidence reveals that both acquired (*de novo*) and pre-existing drug resistance mechanisms exist. For example, pre-existing mutations in MEK1 drive resistance to BRAF inhibitors in melanoma patients [29], whereas acquired mutations in KRAS mediate resistance to Cetuximab, a monoclonal antibody that targets EGFR [30]. Mathematical analysis of the evolution of resistance mutations to imatinib suggested that at most a single clone of resistant mutant cells exists prior to treatment [31]. Earlier reports have indeed reported on resistance single nucleotide variation (SNV) mutations that pre-exist treatment with imatinib [32, 33, 34, 35]. Such mutations can be favorable, neutral, or slightly deleterious in the context of tumor growth. Following on the exponential growth doubling time of transfected murine bone marrow cells revealed that three Abl1 resistance mutations (T315I, E255K and Y253F) grew faster than wild type cells under the experimental conditions [36]. Kinase efficiency was higher than wild-type for two Abl1 mutants (E255K and Y253F) but not for the multi-drug resistance T315I or any of the other tested mutations [36]. Clinical data from imatinib-treated CML and acute lymphoblastic leukemia (ALL) patients revealed preference for the mutants that confer higher kinase activity (E255K and Y253F) [37]. Analysis of the multiple sequence analysis of Abl1 and its homologues reveals that lysine is more common than glutamate and phenylalanine is more common than tyrosine in the positions corresponding to Abl1 E255 and Y253, respectively, which also indicates that these mutations at these positions are favored [25]. Finally, there is evidence of resistance mutations that disappeared upon discontinuation [38] or modification of treatment [39, 40], which indicates that they are somewhat deleterious when they do not confer resistance. The sensitive ultra-deep sequencing methods used in the reported studies [40] revealed that old mutations were wiped out by *de novo* resistance mutations. Altogether, it appears that both acquired and preexisting mutations exist, and that many resistance mutations are deleterious to the tumor, and are not tolerated in the absence of therapy. The latter conclusion may indicate that alternating between therapies can be beneficial.

### Drug resistance is a consequence of endogenous defense mechanisms

Drug resistance can be seen as a necessary evolutionary consequence to the body's need to get rid of toxins [41] or xenobiotics. Protection mechanisms involve proteins that pump the drugs out of the cells (e.g., P-glycoprotein), mutation of the target of a particular toxin, and activation of alternate biological pathways instead of the one hit by a toxin. These are also well-described resistance mechanisms. The mutator phenotype of many cancers, by which they have a much higher rate of mutations compared to benign cells, may also be an endogenous response, since a high mutation rate may accelerate the ability of cells to adapt to a harmful substance [42]. For this reason, it is challenging to deal with drug resistance. In fact, drug resistance is observed in the clinic not only in cancer and pathogen-driven diseases, but also in other cases. One such example is resistance to platelet inhibitors [43], where over-expression of the drug target is one of the mechanisms that lead to failure of treatment.

## THE CANCER STEM CELL HYPOTHESIS

The cancer cell population is very heterogeneous. Cells within a tumor are not equivalent, neither structurally nor genetically [44]. Somatic mutations arise before and during the development of the tumor and lead to evolution of clones of tumor cells, with a different genetic background. The heterogeneity of cancers presents an obstacle to targeted treatment [45] even if the tumor becomes more heterogeneous at times due to selection of the fitter clones (homogenization) [46].

According to the cancer stem cell hypothesis, the bulk tumor cells lack the capacity to self-renew. Only a minority of the cancer cells, namely cancer-stem cells (CSCs), is capable of self reproduction. The stem cells are thus the source of heterogeneity in the tumor. CSCs are rather insensitive to chemotherapy and radiation, can reside in a dormant state for a long duration and can spread to parts of the body other than where they originate from [47]. They thus support three hallmarks of cancer [48]: evading apoptosis, limitless replicative potential and tissue invasion as well as metastasis. The CSCs drive tumorigenesis and drug resistance.

It is not clear how many types of cancer follow the CSC concept, but it is clear that these include leukemias (most notably chronic myeloid leukemia) as well as various solid tumors. The frequency of CSCs among tumor cells varies between different cancers and probably also within tumors. Studies report on frequencies of 10^−6^ in acute myeloid leukemia [49], and 10^−4^ of in solid tumors [50]. In estimating the frequencies of CSCs, it is assumed that they express specific markers (e.g., CD38^−^ CD44^+^ [51]). Of note, the real proportion of cells displaying a CSC phenotype is likely to be higher than reported (though still rather small). This is because there may be multiple types of CSCs within a tumor, each conveying a different set of markers, whereas assays typically search for a specific combination of stem-cell markers. It had previously been noted that “the question of whether the entire neoplasm or a minority of neoplastic cells is capable of self-renewal is, at least in part, a question merely of the effective population size of the evolving cells in a neoplasm” [52]. The effective population of cells capable of self renewal appears to be very large in advanced cancers, whereas it is much smaller in the initial stages of cancers that follow the CSC concept [53].

The CSC hypothesis provides explanation to intriguing phenomena that are observed in many types of cancer. The first of this is recurrence. Patients after treatment may appear and feel healthy again. They show no sign of any neoplasm in medical examinations, yet the disease emerges again after months or even years. This has been explained through the presence of dormant CSC that become active again. Metastases that appear years after a successful surgery are also attributed to dormant CSC. In addition, the CSC hypothesis explains why some cancer cells are able to form tumors when transplanted, whereas others can not [54] - CSCs are tumorigenic whereas differentiated cells are not.

In spite of increasing experimental evidence, the CSC hypothesis remains controversial. From an evolutionary point of view, it is certainly challenging to explain. Why are cancer stem cells rare, given that if all cells had the CSC phenotype cancers would apparently develop faster [55, 56]? One explanation could be that there is actually an evolutionary advantage in dividing the tumor into stem-cells and differentiated cells, as the latter type could carry out more specialized assignments within the tumor such as scavenging nutrients or communicating with the stroma. Furthermore, maintaining a relatively small population of cells that are capable of self-renewal (smaller effective population size) can lead to faster adoption of new traits [57], such as drug resistance.

## CANCER STEM CELLS AND DRUG RESISTANCE

If all cells within a tumor are able to divide and inherit genetic changes to the next generation, there is a strong selection pressure for the cells to develop resistance - those that are not fit will not survive. A crucial difference in cancers that follow the CSC hypothesis is that conventional therapies have a much smaller capacity to kill the CSCs [58, 59, 60, 61]. If resistance mutations preexist treatment, it is clear that only descendants of resistant CSCs will survive and out-compete all other cells. For resistance mutations to occur *de novo*, new mutations have to arise. Indeed, data from leukemia patients suggests that tumor heterogeneity increases significantly following treatment, *i.e*., quite often not one but several different resistance mutations occur [40]. These mutations are not observed prior to treatment even with state-of-the-art sequencing techniques that are very sensitive. The onset of multiple mutations could be explained by an increase of the mutation rate of the CSCs, by an increase in the number of cell divisions, or by a combination of the two. The biological mechanism of compensatory proliferation is in line with the second mechanism and could explain how resistance emerge within cancers that follow the CSC hypothesis.

## THERAPEUTIC RESPONSE AND PATIENT SURVIVAL

An important limitation of the discussion on the fitness of tumors is that the subject of treatment is not the tumor but the patient. Therapeutic response (diminishment of the tumor or progression free survival) does not necessarily translate to overall survival [62]. The CSC theory alone cannot explain this paradox. At the very least, therapy should prolong the life of the patient by as long as the progression free survival phase, but the benefit (in terms of life expectancy) of therapy is often smaller [63]. It is not that targeted therapy is ineffective. It is often capable of shrinking the tumor and in many cases increases overall survival. Imatinib and other Bcr-Abl agents, for example, can increase the life-expectancy of patients by many years. Some newly approved KIs such as afatinib also carry out significant benefits in terms of overall survival (12 month benefit relative to cisplatin according to the manufacturer), whereas the situation is less clear with others. On December 2015, The European Medicines Agency recommended the EGFR inhibitor osimertinib for conditional approval, while noting that “The benefits in terms of progression free survival and/or overall survival have not yet been determined”. Other agents failed in clinical trials due to inability to improve the overall survival. Some recent examples include the mTor inhibitor everolimus, which did not improve overall survival in hepatocellular carcinoma patients after failure of sorafenib [64]; and bevacizumab, which did not improve the overall survival in first-line treatment for glioblastoma [65].

Natural selection within the tumor can be used to explain the inability of some drugs to increase the overall survival despite (temporarily) limiting tumor growth. The drugs select for the most resilient cells, which often leads to a more rapidly increasing and metastatic tumor (oncogenic resistance [16, 63, 66]). Moreover, diminishment of the effective population size increases the probability of fixation of novel mutations (*vide supra*, “Pre-existing or acquired resistance”), which may increase the fitness of the tumor relative to the pre-treatment state. Another contributing factor is that side effects of the treatment often weaken the patient and limit the ability of body to survive the disease. Furthermore, depending on the activity of multiple signaling networks, a given therapy may be totally inadequate in a particular stage [9, 10]. Finally, compensatory proliferation may also partially explain why overall survival is shorter than expected.

## COMPENSATORY PROLIFERATION

To rationalize the evolution of multiple *de novo* resistance mutations, we invoke the concept of compensatory proliferation. Compensatory proliferation is discussed mainly in the context of developmental biology. Simply put, this concept states that dying cells (usually through apoptosis), convey signals to the nearby cells thereby stimulating mitosis [67]. This results in (partial) compensation for the death of cells. Such compensatory mechanisms were discussed for the first time in a study of fruit flies that survived and had normal appearance in spite of having been exposed to radiation [68]. A compensatory mechanism, where the surviving cells were stimulated to proliferation was proposed to explain the findings.

**Figure 3:**
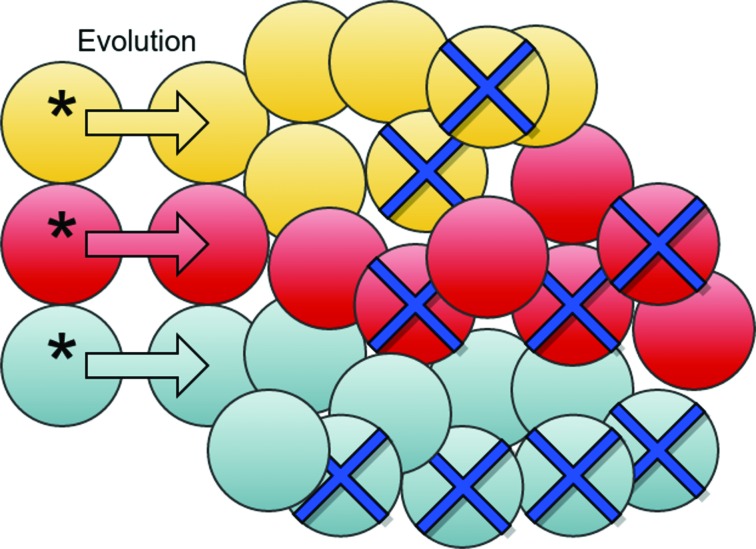
Cancer stem cells and drug resistance CSCs (marked with an asterix) can undergo mutations during replication and thereby drive evolution in the bulk tumor. The size of the tumor diminishes upon targeted treatment due to dying cells (marked with crosses), but CSCs are less sensitive to the treatment.

**Figure 4:**
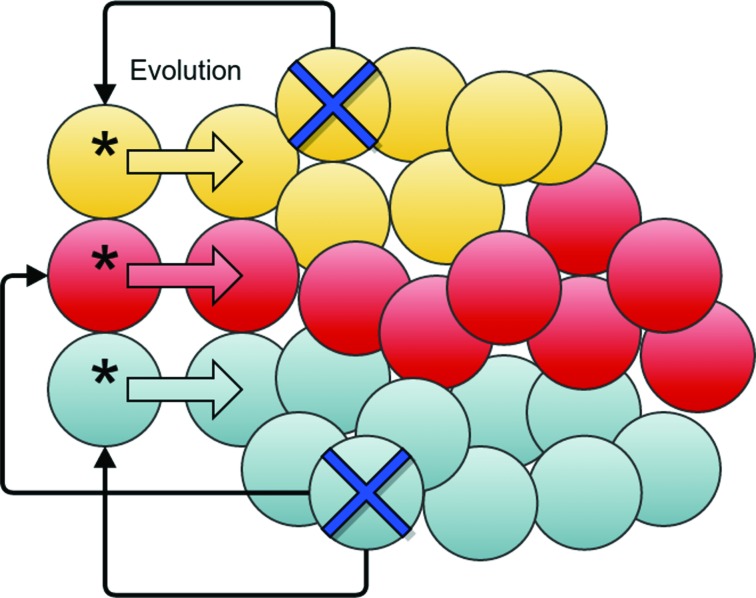
Cancer stem cells and compensatory proliferation Dying cells (marked with crosses) send signals (black connector arrows) to the CSCs (marked with asterix). The CSCs increase their replication rate, which leads to new mutations. Some of these mutations can be beneficial to the tumor's fitness and can thus become prevalent in the population.

Targeted treatment kills the bulk of tumor cells, but is much less effective against CSCs (Figure 3). Compensatory mechanism provokes self-renewal of CSCs (Figure 4). The rate of self-renewal (birth rate) is lower than the death rate of sensitive cells, and hence the tumor shrinks. Since mutations are generated through meiosis, gene replication, recombination and chromosome segregation, an increased rate of CSC replication leads to an increased rate of mutations. The vast majority of the mutations that survive will be nearly neutral, but occasionally a resistance mutation will emerge. Thus, compensatory proliferation explains the development of resistance within cancers that follow the CSC hypothesis, even if “stem cells are resistant to resistance” [63] (as they are not sensitive to targeted therapy).

Do we need to account for compensatory proliferation to explain the evolution of drug resistance? Mathematical reasoning led to the conclusion that resistance mutations predate treatment but become fixed in the population following targeted therapy [69]. While this corroboration explains many earlier findings, it is at odds with recent evidence that showed a large proportion of various mutations in drug targets following treatment. Another puzzling finding that can be explained through compensatory mechanism is a sudden increase in the number of mutations in the drug target Abl1 following change of drugs [40]. The Abl1 kinase is the primary target for treating Ph+ leukemias. Different Abl1 inhibitors are used in the clinic, with various resistance profiles. When resistance to a given Abl1 inhibitor is observed, a patient is often advised to switch to a different drug. Such changes can result in temporary relief, but relapse often follows from development of new resistance mutations. Interestingly, it has been found that many of the patients initially develop several different mutations, each covering a sub-clone of the tumor [39, 40]. Genetic modifications are observed in as little as few weeks after treatment, but the mutations are not observed until the new treatment initiates, even when using the most sensitive techniques [39]. Thus, it is unlikely that resistance mutations emerged prior to treatment, and more likely that mutations emerge as a result of the targeted therapy (*de novo* mutations). This observation can be explained by a compensatory mechanism that leads to an increase in number of cell divisions and hence mutations.

### Biological mechanism

The most well described compensatory mechanism is apoptosis-induced compensatory proliferation (AICP). Cytotoxic cancer treatments (chemotherapy and radiation) are known to induce apoptosis. How targeted therapy kills tumor cells is less clear. There is some evidence, how-ever, that drugs such as imatinib lead to elevated levels of activated caspases [70], which are apoptosis-related enzymes. Likewise, challenging NSCLC lines with the kinase inhibitor erlotinib activates caspases [71]. Targeting apoptotic pathways is an active line of development of cancer therapies [72, 73].

Caspases are central to the process of apoptosis and to AICP. Their interactions with down-stream effectors lead to cell death and to expression of mitogens. The mitogens in their turn diffuse to nearby cells or compartments and induce mitosis of CSCs. Two mechanisms of AICP are known in Drosophila Melanogaster. One of these involves the JNK signaling pathway [74]. Interestingly, studies reveal JNK proteins to have a dual role: they can be either proto-oncogenes or tumor suppressors [75]. The first role may be due to stimulation of proliferation of CSCs, whereas tumor suppression may stem from JNK involvement in apoptosis. Other than JNK, calcium-independent phospholipase A2 (iPLA2) seems to be important for AICP in mice [76]. Interestingly, in spite of its role in mediating apoptosis, iPLA2 was also indicated as a drug target for ovarian cancer, due to its role in promotion of proliferation [77].

Recently, a different compensatory mechanism has been proposed based on experiments in bladder tumors [78]. In these tumors, dying cells release a signaling lipid molecule called prostaglandin E2 (PGE2). PGE2 leads to proliferation of cells, similarly to repopulation of a tissue after injury. It has been shown that drugs that block the synthesis of PGE2 inhibit the repopulation of the tumor. This suggests that such treatments can also limit the development of resistance mutations.

## CONCLUSIONS

Understanding how resistance to targeted therapy occurs is necessary to prolong the effect of modern anti-cancer drugs. This is not as straightforward as in communicable diseases, where it is clear that the drug kills the pathogen and therefore the pathogen must develop resistance to survive. In cancers, the principle of compensatory proliferation is used to explain how resis-tance to therapy occurs, in particular in neoplasms that follow the cancer stem cell hypothesis. This calls for a better characterization of compensatory mechanisms in mammals. Two pathways seem to be involved in AICP according to the current knowledge: JNK and iPLA2. Thus, it may be possible to use combination therapy that blocks the traditional drug target (such as a kinase or a hormone receptor) and mitogens that are responsible for proliferation through signaling. The discovery of selective inhibitors of JNK enzymes [79] shall enable the testing of dual-inhibition scheme and can reveal its pros and cons. In some cancers, PGE2 is involved in tumor cell repopulation [78]. Given that commonly used non-steroidal anti inflammatory drugs inhibit the synthesis of PGE2, it may soon be possible to test whether a combination of such drugs and targeted therapy or chemotherapy can circumvent drug resistance.

Theory [80] and simulations [81] predicted that drugs targeting cooperation among individuals in bacterial colonies will be less prone for development of resistance. This has recently been demonstrated in an experiment, where bacteria were subject either to gallium or to antibiotics [82]. The gallium ions targeted sidosphores, metal-chelating molecules that are secreted by bacteria to scavenge iron ions. Since sidosphores are secreted, they improve the survival of the colony but do not necessarily increase the fitness of the bacterial cells that secret them. Indeed, resistance was observed against antibiotic treatment but not against treatment with gallium ions. Likewise, compensatory proliferation mechanisms may be useful as a drug target in cancers, and resistance to their inhibitors is likely to be more limited than to targeted therapies.
